# Effect of berrycactus fruit (*Myrtillocactus geometrizans*) on glutamate, glutamine, and GABA levels in the frontal cortex of rats fed with a high-fat diet

**DOI:** 10.1515/biol-2022-0529

**Published:** 2023-01-24

**Authors:** Cuauhtémoc Sandoval-Salazar, Sandra Neli Jiménez-García, Vicente Beltrán-Campos, Luz Elvia Vera-Becerra, Carlos Alberto Núñez-Colín

**Affiliations:** Division of Health Sciences and Engineering, University of Guanajuato, Campus Celaya-Salvatierra, Celaya 38060, México; Division of Health Sciences, University of Guanajuato, Campus León, León 37670, México

**Keywords:** berrycactus, high-fat diet, glutamate, glutamine, GABA

## Abstract

In addition to the known metabolic alterations, obesity has consequences at the brain level, driving imbalance in neurotransmitters such as glutamate (Glu), glutamine (Gln), and gamma-aminobutyric acid (GABA). The consumption of fruits with antioxidant properties, such as the berrycactus *Myrtillocactus geometrizans*, could have beneficial effects in such an imbalance. The study objective was to evaluate frontal cortex neurotransmitter levels and weight changes in rats fed with a high-fat diet (HFD) and *MG*. To achieve that, five groups of Wistar rats received different diets for 24 weeks: standard diet (SDt), HFD, HFD + *MG* extract 150 mg (HMg150), HFD + *MG* extract 300 mg (HMg300), and HFD + *MG* extract 450 mg (HMg450); rats received *MG* extract for the last 4 weeks. Weight and food intake were recorded every week, and also neurotransmitter levels were quantified using high-performance liquid chromatography. Groups fed with HFDs had increased Glu and Gln levels, decreased GABA, and also gained more weight compared to the SDt group; *MG* extract of 450 mg decreased Glu levels. Concentrations of 300 and 450 mg of *MG* extract decreased weight compared to the HFD and HMg150 groups. This study reports that HFDs have an impact on neurotransmitter levels and weight, *MG* extract showed a reduction in Glu concentration and weight.

## 1 Introduction

Obesity is a chronic metabolic condition that is associated with the development of cardiovascular diseases and metabolic disorders such as type 2 diabetes mellitus, dyslipidemia, and hypertension [1]. The origin of this condition is influenced by eating habits and genetic and neurobiological factors; evidence shows that obesity is associated with increased vulnerability to brain damage [2] due to the consumption of high-fat diets (HFDs) [3].

The accumulation of visceral adipose tissue can affect the metabolism of the neurons and change the cycle and synthesis of the neurotransmitters glutamate (Glu), gamma-aminobutyric acid (GABA), and glutamine (Gln) (Glu/GABA-Gln). In this cycle, neurons need to release Glu and GABA, then those join the postsynaptic cells and after recapture with the participation of astrocytes, which in turn release Gln; it will be necessary for the synthesis of new neurotransmitters in the presynaptic neurons [4]. For this process, adequate levels of glucose are needed that are obtained from the bloodstream through astrocytes [5]; however, due to the consumption of high saturated fat diets, it seems that the levels of neurotransmitters are modified [6]. In this regard, it has been shown as a metabolic decrease in the glycolytic activity in the Krebs cycle in the brain, with changes in the levels of neurotransmitters such as Glu and Gln [7]. In addition, in earlier work, we found that the consumption of a HFD decreases GABA levels [8] and alters the functionality of the enzyme glutamic acid decarboxylase and Gln synthase in the hippocampus and cortex. These modifications could be due to lower glucose levels that affect the Krebs cycle and its intermediate products such as α-ketoglutarate [9], as well as the oxidative stress produced by HFDs.

Specialist recommend consumption of functional and accessible foods, including Berries. These fruits provide few calories, fiber, and antioxidant compounds, such as vitamins A and C, flavonoids, and tannins, and act at the brain level decreasing oxidative stress and reducing proinflammatory cytokines [10].

Mexico occupies the first place worldwide in overweight and obesity, which have been related, among other factors, to the consumption of foods high in saturated fats. The consumption of foods rich in antioxidants could help to prevent oxidative damage in proteins, lipids, carbohydrates, and deoxyribonucleic acid caused by the intake of foods high in saturated fat. Intake of phenolic compounds may reduce oxidative stress caused by chronic diseases [11], food products such as red wine has shown a protective effect, increasing the survival of Saccharomyces cerevisiae yeast cells, stressed with H_2_O_2_ [12]. Other functional foods with antioxidant activity due to the phenolic compounds and anthocyanins are strawberries [13] and *Myrtillocactus geometrizans* [Garambullo or berrycactus fruit] (*MG*), which has polyphenols and flavonoids with antioxidant capacity [14]. *MG* has carbohydrates, proteins, macronutrients, such as N and K, micronutrients such as Fe, Zn, and Mn, and also polyphenols and flavonoids [15].

In this regard, *MG* decreases glucose and triglyceride levels, as well as reduce oxidative stress by increasing the enzymatic activity of glutathione among diabetic mice [16]. Similarly, it has been found that in cultures of cancer cell lines, doses of 100 mg/kg of *MG* inhibits inflammation and reduces the viability of cancer cells [17], and shows apoptogenic effects *in vitro* [18]. However, the *MG* effect in the brain is unclear.

The aim of the present study was to evaluate frontal cortex neurotransmitter levels and weight changes in rats fed with a HFD and *MG*.

## 2 Methods

### 2.1 Experimental animals

Twenty-five 1 month old male Wistar rats (100 g weight) were kept in a polypropylene animal cage in a temperature-controlled environment (22 ± 2°C), and under a light–dark cycle set at 12:12 h inside an animal facility at the University of Guanajuato.


**Ethical approval:** The research related to animal use has been complied with all the relevant national regulations and institutional policies for the care and use of animals and has been approved by Bioethics Committee, Campus Celaya-Salvatierra at the University of Guanajuato and the National Research Council Guide for the Care and Use of Laboratory Animals and the Official Mexican Regulation for Experimentation in Animals (NOM-062-ZOO-1999).

### 2.2 Treatment

Rats were acclimatized to their environment for 1 week and randomized into five groups (*n* = 5), to receive the following treatments: (1) Standard diet group (SDt), (Purina Rodent Chow; Purina Mexico: protein 23%, fat 4.5%, and carbohydrates 72.5%), (2) HFD group, (Purina Chow; Purina Mexico: protein 12.1%, fat 20% + 20% lard added, and carbohydrates 21.3%), (3) HFD + *MG* extract 150 mg group (HMg150 mg), (4) HFD + *MG* extract 300 mg group (HMg300 mg), and (5) HFD + *MG* extract 450 mg group (HMg450 mg). The *MG* fruits were washed in clean water and were maintained for 24 h at −50°C and 0.012 atm in a vacuum system, then the fruits were weighed to know the product left after the drying process. For phenol extraction, 25 mg of dry samples were taken, and 2.5 mL of methanol was added to each sample, which were then kept away from light and were shaken for 24 h. The sediment formed at the bottom was then centrifuged at 5,000 rpm for 10 min at 4°C, and the supernatant was removed. The total phenol content was determined by the Folin–Ciocalteu spectrophotometric method modified for use in 96-well microplates. In quantification, an aliquot of the methanolic extract (4 µL) was mixed, 250 µL was mixed in the Folin–Ciocalteu reagent (1N), 1,250 µL of Na_2_CO_3_ (20%) were added and kept in the dark for 2 h at room temperature. Subsequently, absorbance at 760 nm was measured in a spectrophotometer (Multiskan GO). The results were expressed in mg gallic acid equivalents/g dry weight. The concentration of total phenols in the berrycactus juice is 142 mg of gallic acid equivalents/100 g [19]. The groups had access to water and chow ad libitum. All the groups were fed for 24 weeks, and HMg150, HMg300, and HMg450 groups received *MG* extracts mixed in water for the last 4 weeks by intragastric administration. Food intake and body weight were recorded each week for the 24 weeks.

### 2.3 Collection of tissue samples

After intervention, the rats were anesthetized with intraperitoneal sodium thiopental (50mg/kg) to obtain blood and tissues. Immediately, the frontal cortex was removed, dissected on ice-cold glass, and stored in microtubes at −80°C until analysis.

### 2.4 Neurotransmitters level measurements

The Glu, Gln, and GABA levels were quantified using high-performance liquid chromatography (HPLC) equipped with a reverse-phase XTerra^®^ C18 column (5 μm, 3 mm × 100 mm) and Waters^®^ 515 pump, O-phthaldialdehyde (OPA) derivatization and electrochemical detection using a BASS-LC 4C system coupled to an amperometric detector. Briefly, frontal cortex (*n* = 5 per group) was defrosted slowly on ice and mixed with cold solution containing equal proportions of methanol and phosphate buffer saline (PBS) of 0.1M and pH 7.4 to prepare homogenates by ultrasonication with a final concentration of 200 mg tissue/mL. Afterward, the homogenates were centrifuged at 13,500 rpm for 20 min at 4°C and the supernatant was recovered and filtered. Standard solutions of the amino acids with concentrations of 5, 10, 25, 50, 100, or 200 μmol/L in PBS were used to prepare a calibration curve. Amino acids were subjected to a derivatization reaction with OPA reagent according to a previous protocol. Then, 10 μL of standard solution or sample (previously diluted 1:2) was mixed with 40 μL of OPA reagent. After 2 min of reaction, the sample was injected into the HPLC apparatus equipped with a reverse-phase Xterra^®^ C18 column (5 μm, 3 Å ∼ 100 mm) and Waters^®^ 515 pump. The configuration consisted of a 0.45 mL/min flow, 15,000 psi pressure, and redox potential of 0.642 V. Amino acids were separated using a gradient program and three mobile phases of methanol 20% (A) and 80% (B) in phosphate sodium (NaH_2_PO_4_) buffer of 0.05 M and pH 5.5. The area under the curve for the standards and samples was determined and used to calculate the concentrations of GABA, Gln, and Glu which were normalized to μmoles/g of wet tissue. All the measurements were conducted at room temperature [7].

### 2.5 Statistical analysis

For the statistical analyses, we used Statistics for Windows 8 (StatSoft, Inc.). Because the lack of homoscedasticity of the data can generate bias in the parametric analysis by ANOVA (analysis of variance), Kruskal–Wallis test was applied followed by a Z-based post hoc test for multiple comparisons [20], expressing the results as the mean value ± standard error of the mean (SEM). The significance level was set at *P* ≤ 0.05.

## 3 Results

### 3.1 Neurotransmitter levels modification on the frontal cortex

Table 1 shows the changes in the levels of neurotransmitters Gln, Glu, and GABA in the frontal cortex of rats fed with a HFD. We do not find differences in the Gln concentration among the HFD group compared to the SDt group. However, we saw significant increases (*P* ≤ 0.05) between the groups treated with *MG*, HMg150, HMg300, and HMg450, when compared to the Gln levels of the SDt group.

**Table 1:** Effect of HFD on neurotransmitters levels

Groups	Frontal cortex neurotransmitter levels (mmol/g/tissue)		
	Gln	Glu	GABA
SD	563.565^a,b^	772.356^b^	325.910^a^
HFD	436.194^b^	1264.732^a^	279.643^a,b^
HMg150	592.251^a^	1288.778^a^	224.775^a,b^
HMg300	591.939^a,b^	1078.295^a,b^	193.087^b^
HMg450	608.813^a,b^	925.899^a,b^	245.900^a,b^

SDt (standard diet); HFD (high fat diet); HMg150 (HFD + *MG* extract); HMg300 (HFD + *MG* extract), and HMg450 (HFD + *MG* extract). Data are reported as the mean value ± SEM. In the columns, values with same letter means no significant differences by Kruskal–Wallis and Z-based *post hoc* rank test at *P* ≤ 0.05.

About Glu, there was a significant increase (*P* ≤ 0.01) in its concentration on the four groups treated with the HFD, HFD group, HMg150, HMg300, and HMg450, compared to the SDt group. For this neurotransmitter, the *MG* concentration of 450 mg was able to decrease Glu levels (*P* ≤ 0.01) compared to the HFD and HMg150 groups. The concentration of GABA levels, in the groups that received HFD, HMg150, HMg300, and HMg450, had a significant decrease (*P* ≤ 0.05) compared to the SDt group. Similarly, a decrease was seen in GABA in the HMg300 group compared to the HFD group (*P* ≤ 0.05), as well as differences between the HMg150 and HMg300 groups (*P* ≤ 0.05), and the HMg300 and HMg450 groups (*P* ≤ 0.05).

### 3.2 Body weight gain during HFD exposure and *MG* treatment

**Figure 1:**
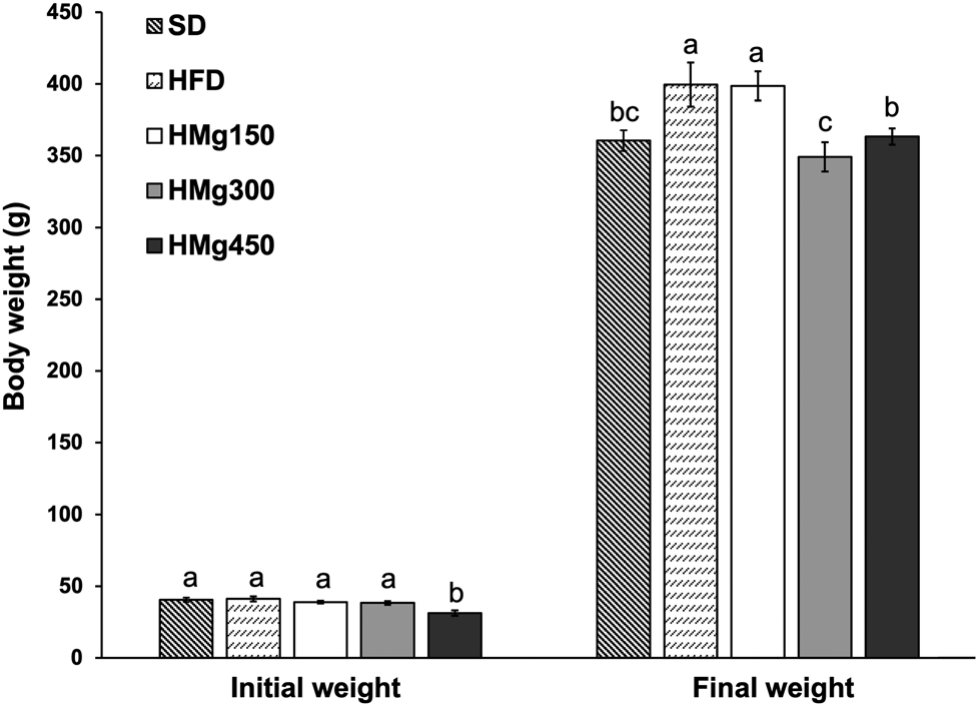
Effect of *MG* on body weight gain, SDt, HFD, HMg150, HMg300, and HMg450. Data are reported as the mean value ± SEM. In the bars, values with the same letter means no significant differences by Kruskal–Wallis and Z-based post hoc rank test at *P* ≤ 0.05.

The rats were weighed before the beginning of the treatment, and no significant differences were seen between the groups (*P* = 0.075), Figure 1. At the end of the treatment, all the groups had a significant increase in body weight compared with the baseline weight (*P* = 0.001). No effects on weight were noted with the *MG* treatment among HMg150 group. However, the HMg300 (*P* = 0.006) and HMg450 (*P* = 0.049) groups significantly decreased in weight compared to the HFD group.

**Figure 2:**
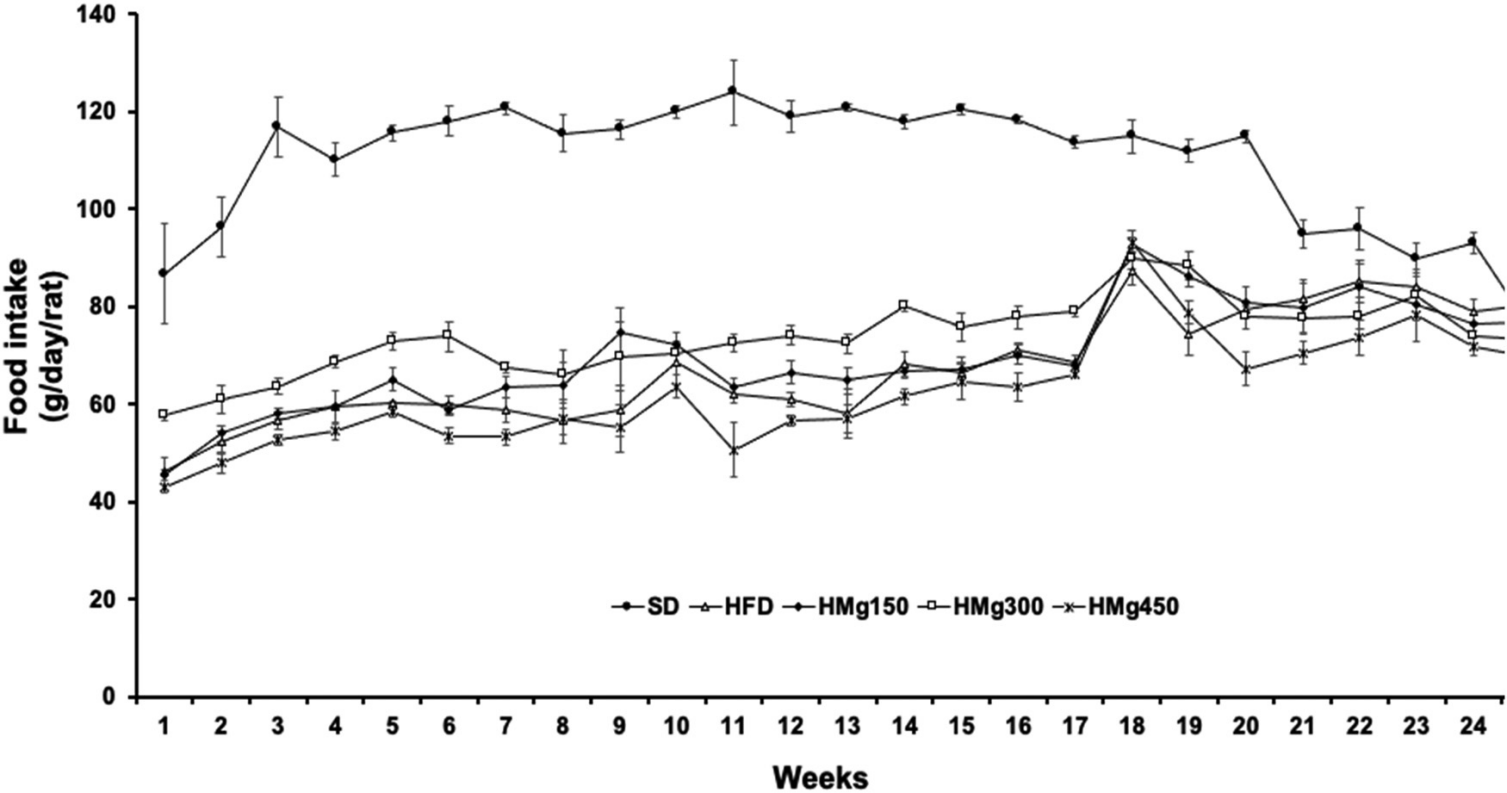
Effect of HFD on food intake during treatment, SDt, HFD, HMg150, HMg300, and HMg450. Data are given as the mean value ± SEM.

In addition, we evaluated dietary intake we; in this regard, any extracts concentration had effect on changing dietary intake. However, dietary intake was lower (30%) in all the groups with a HFD compared to the SDt (Figure 2).

## 4 Discussion

In this study, we found that the administration of HFD for 24 weeks increased the levels of the Gln and Glu and decreased the concentration of GABA in the frontal cortex. The *MG* extract of 450 mg was able to reduce the concentration of Glu levels and the weight of the rats fed with HFDs.

It has been reported that inadequate nutrition not only influences the development of overweight and obesity, but also has an effect on brain processes that involve neuronal communication through neurotransmitters, mood, and cognition [21]. Specifically, Western diets have high concentrations of saturated fats, and those diets produce inflammation, affecting cognitive processes [22]. In the present study, HFDs and three concentrations of *MG* extract were used, and it was found that HFDs increased Gln and Glu levels in the frontal cortex but decreased the concentration of GABA. Other reports have shown that the consumption of HFD in rats decreases the concentration of serotonin, while it increases dopamine and Glu levels [6].

### 4.1 Berrycactus and Glu levels in the frontal cortex

In the brain, the synthesis of neurotransmitters takes place between the interaction of neurons and glial cells such as astrocytes, which play an essential role in the Glu/GABA-Gln cycle. This highly regulated process requires the participation of transporters for each neurotransmitter [4]. For this, the consumption of a balanced and sufficient diet is essential, otherwise, HFDs increase ammonium levels and mitochondria metabolism, and then the Krebs cycle and the regulation of neurotransmitter synthesis can be affected [23]. The increase in Glu found in the groups treated with HFDs coincides with a work carried out in 2020, in which, rats fed with HFD for 4 weeks had increased levels of Glu and dopamine [6]. Similarly, the administration of western diet for 24 weeks altered the glutamatergic functionality of medium dorsolateral spiny neurons, with attribution to a deficiency in its recapture [24]. Also, prolonged consumption of HFDs induces a decrease in vesicular transporters to Glu [24], increases Glu metabolism, affects the Gln-Glu cycle and increases other molecules such as aspartate and choline [25]. However, in the present study, the concentration of 450 mg of *MG* extract decreased Glu levels, the consumption of this fruit could have beneficial effects and help to counteract the adverse effects of HFDs. This may be due to its polyphenolic and flavonoid antioxidant components [15]. The increase in Glu levels could produce excitotoxicity [26]) and so affect the synthesis processes of other neurotransmitters such as Gln and GABA, as well as various cognitive processes.

### 4.2 HFD modifies the Gln concentration

There is a direct interaction between Glu and Gln in the brain, once Glu is released at the synapse, it is rapidly taken up by astrocytes and converted to Gln by Gln synthase (GS) allowing its rapid removal as part of the Glu-Gln cycle [26]. However, deficiency in its reuptake is attributed to the loss or alteration of the function of the astrocytic transporter GLT-1. The presence of Glu in the synaptic cleft is kept for a longer time and so a greater stimulation is produced in the post-synaptic neuron [24]. Therefore, in the present investigation, the increase in Glu found in the groups fed with HFD may be due to a deficiency in its recapture and degradation by astrocytic GS. This gives importance to astrocytes in protecting ammonia toxicity neurons from ammonia toxicity, Glu excitotoxicity, and its GS-dependent conversion to Gln, as well as due to a deficiency in astrocytic GS, which is altered by the increase in ammonia produced by the chronic consumption of HFDs [26]. It was found that *MG* did not have an effect to reduce this neurotransmitter, this could be due to an imbalance in the Gln/Glu cycle ratio, given by high Glu levels; therefore, nutrition interventions, as is treatment with *MG,* is needed for longer, since HFDs for extended periods of time generate neuroinflammation. In this regard, a study found that an increase in the microglial activity of obese rats, after treatment with sour cherries for 17 weeks, led to a reduction in the activity of the glial fibrillary acidic protein [27].

### 4.3 Saturated fats consumption decreases GABA levels

Under normal conditions, the synthesis of GABA through GAD requires its precursor Glu. However, earlier studies have seen that the consumption of HFD decreases its levels in structures such as the frontal cortex and hippocampus [28]. These alterations could be due to oxidative stress and neuroinflammation produced by the consumption of HFDs [3]. In the present study, we found that HFDs decreased GABA levels in the frontal cortex. To keep this neurotransmitter at adequate levels, a balance in the Glu/GABA/Gln cycle is needed [4], and for this, the quality of the diet could be a key factor, since the consumption of HFDs changes both, the levels of neurotransmitters and the vesicular transporters that take part in the synaptic function [24]. Although not significant, *MG* 450 mg extract tended to raise GABA levels compared to the other groups, in other investigations conducted by our group, we evaluated the use of strawberries irradiated with ultraviolet light for 12 weeks, and we found that strawberries increased GABA levels in the frontal cortex [8]. Therefore, treatment time may be a key factor in restoring cellular homeostasis in the brain using antioxidant treatments such as the *MG*. Therefore, this could say that the antioxidant components of the berrycactus exert effect at the cellular level; however, we did not characterize or evaluate polyphenols and antioxidants separately. Thus, nutrition is a crucial factor in keeping the brain in best condition. For example, rats fed with rice enriched with GABA show decreases in the levels of cell damage produced by oxidative stress generated by HFDs [29].

### 4.4 Antioxidant fruits and overweight regulation

Similarly, in this study, groups treated with HFD showed greater weight gain compared to the SDt group. In this regard, HFD not only increases body fat mass [30] but also reduces synaptic plasticity in the hippocampus and cerebral cortex. In the present study, the groups treated with concentrations of 300 and 450 mg of *MG* decreased in weight. This could be due to that *MG* has 68% carbohydrates in its nutritional composition [15], and therefore, a rapid change in flavor could produce an effect and reduce food consumption favoring weight loss [30]. Food intake involves processes as appetite, motivation, energy requirement, as well as the availability, exposure, and quality of food [31]. The present study found that the HFD groups consumed less food but managed to gain weight, fat is an important contributor to total energy intake and satiating properties that could potentially reduce energy intake [32]. It has also been seen that the consumption of berries may not affect weight loss; those results are not conclusive and, although seems that antioxidant compounds such as flavonoids have benefits in insulin resistance, dyslipidemia, and hypertension, it would be necessary for more studies to find its direct effects on weight [27].

Our results show that the HFDs change the concentration of neurotransmitters by increasing the levels of Glu and Gln and decreasing those of GABA; interestingly, the *MG* extract reduces Glu levels. These outcomes suggest that the consumption of this berrycactus could be a strategy to regulate neurotransmitters and improve brain processes affected by the consumption of HFDs.
